# Trends and Costs Associated With Suboptimal Physical Activity Among US Women With Cardiovascular Disease

**DOI:** 10.1001/jamanetworkopen.2019.1977

**Published:** 2019-04-12

**Authors:** Victor Okunrintemi, Eve-Marie A. Benson, Martin Tibuakuu, Di Zhao, Oluseye Ogunmoroti, Javier Valero-Elizondo, Martha Gulati, Khurram Nasir, Erin D. Michos

**Affiliations:** 1 Department of Internal Medicine, East Carolina University, Greenville, North Carolina; 2 Department of Epidemiology, Johns Hopkins Bloomberg School of Public Health, Baltimore, Maryland; 3 Ciccarone Center for the Prevention of Cardiovascular Disease, Johns Hopkins School of Medicine, Baltimore, Maryland; 4 Department of Medicine, St. Luke’s Hospital, Chesterfield, Missouri; 5 Center for Outcomes Research and Evaluation, Yale New Haven Hospital, New Haven, Connecticut; 6 Division of Cardiology, University of Arizona College of Medicine, Phoenix; 7 Division of Cardiology, Yale School of Medicine, New Haven, Connecticut

## Abstract

**Importance:**

Cardiovascular disease (CVD) is the leading cause of death and disability among women. Achievement of recommended physical activity (PA) levels is an essential component of CVD management.

**Objective:**

To describe trends, sociodemographic factors, and health care expenditures associated with suboptimal PA among a nationally representative sample of US women with CVD.

**Design, Setting, and Participants:**

This cross-sectional study used serial data from the Medical Expenditure Panel Survey from 2006 through 2015. The analyses were conducted in August 2018. Women who had self-reported and/or *International Classification of Diseases, Ninth Revision*, diagnosis of CVD were included.

**Main Outcomes and Measures:**

*Recommended PA* was defined as 30 minutes or more of moderate- to vigorous-intensity exercise, 5 or more days per week. Weighted logistic regression was used to examine the associations of various sociodemographic factors with suboptimal PA, adjusted for comorbidities. A 2-part econometric model was used to assess health care expenditures.

**Results:**

A total of 18 027 women were included in this study. The results were weighted to provide estimates for approximately 19.5 million adult women in the United States with CVD (mean [SD] age, 60.4 [16.9] years). More than half of the women with CVD reported suboptimal PA, a trend that increased during the 10-year period, with 58.2% (95% CI, 55.9%-60.5%) of participants reporting suboptimal PA in 2006-2007 vs 61.9% (95% CI, 59.7%-64.2%) in 2014-2015 (*P* = .004). The proportion of women with suboptimal PA differed by sociodemographic factors. In adjusted models, compared with non-Hispanic white women, African American women (odds ratio, 1.22; 95% CI, 1.08-1.38) and Hispanic women (odds ratio, 1.33; 95% CI, 1.13-1.58) were more likely to have suboptimal PA. Women from low- or very low-income strata (compared with high-income strata), enrolled in public insurance (compared with private insurance), and with less than high school education (compared with at least some college education) were more likely to have suboptimal PA. Health care costs among women with CVD with suboptimal PA were higher compared with those among women who met the recommended PA, and this increased through time, from a mean total health care expenditure of $12 724 (95% CI, $11 627-$13 821) in 2006-2007 to $14 820 (95% CI, $13 521-$16 119) in 2014-2015.

**Conclusions and Relevance:**

The proportion of women with CVD not meeting recommended PA is high and increasing, particularly among certain racial/ethnic and socioeconomic groups, and is associated with significant health care costs. More must be done to improve PA for secondary prevention and reduction of expenditures among women with CVD.

## Key Points

**Question:**

What are the trends and health care expenditures associated with not meeting recommended physical activity (PA) levels among a representative sample of US women with cardiovascular disease?

**Findings:**

In this cross-sectional study of 18 027 women, using 10-year data from the Medical Expenditure Panel Survey, more than half of women had suboptimal PA, with higher proportions among subgroups defined by age, race/ethnicity, and socioeconomic factors. The economic burdens associated with suboptimal PA were higher compared with optimal PA.

**Meaning:**

Specific interventions targeting older women, lower socioeconomic status, and racial/ethnic minorities should be implemented to enable more women to achieve optimal PA for secondary prevention and reduction in health care costs.

## Teaser

This cross-sectional study uses Medical Expenditure Panel Survey data to describe trends and health care expenditures associated with physical activity levels among women with cardiovascular disease.

## Introduction

Cardiovascular disease (CVD) is the leading cause of death and disability among women in the United States.^1^ The landmark INTERHEART study^2^ suggested that 90% of excess coronary risk might be attributable to 9 modifiable risk factors, including regular physical activity (PA). Thus, it is imperative to direct more attention toward the prevention and management of traditional risk factors, with special attention to lifestyle interventions in women.^3^

Lack of regular PA has been independently linked to a higher risk of CVD, obesity, diabetes, and all-cause mortality.^4,5^ Thus, exercise should be viewed as a preventive medical intervention. The American Heart Association^6^ and the US Department of Health and Human Services^7^ recommend the achievement of at least 150 minutes per week (typically through ≥30 minutes per day, ≥5 days per week) of moderate- or vigorous-intensity activity. Despite these recommendations, which have been in place for the last 2 decades, prior studies have indicated that PA declines with age^8^ and that women are, overall, less physically active than men at all ages.^9^

Numerous studies have demonstrated a clear and significant benefit of regular PA in high-risk secondary prevention populations.^10,11^ Patients enrolled in exercise-based cardiac rehabilitation programs have lower risk of reinfarction, reduced hospitalization rates and mortality, better exercise performance, and improved health-related quality of life compared with those not enrolled.^12,13,14^

Although the economic impact of PA on overall well-being has been widely studied,^15,16,17,18,19,20^ relatively few of these studies focused on women, to our knowledge. Our aim was to describe the trends, predictive factors, and health care expenditures associated with suboptimal PA levels among a nationally representative sample of US women with CVD from January 1, 2006, to December 31, 2015.

## Methods

### Study Population and Survey Years

We used retrospective data from the Medical Expenditure Panel Survey (MEPS)^21^ spanning 10 years, from January 1, 2006, to December 31, 2015. The MEPS is a national survey of individuals, families, and health care personnel and provides information on sociodemographic characteristics, medical conditions, prescription medications, patient experiences, health resource utilization, and health care expenditures.^22,23^ The MEPS is cosponsored by the Agency for Healthcare Research and Quality and the National Center for Health Statistics. The MEPS collects data from a nationally representative sample of households using an overlapping panel design in which a new panel of households is chosen yearly and information from each panel is collected in 5 rounds of interviews in 2 calendar years. This design serves to provide continuous and up-to-date health care expenditure estimates per calendar year.^23,24^ Agency for Healthcare Research and Quality researchers assign person-weight and variance estimation stratum to each participant to reflect survey nonresponse and population sums. Details of the data collection process have been described by the Agency for Healthcare Research and Quality.^24,25^

For this study, the MEPS Household Components full-year consolidated files, which contain information on sociodemographic characteristics and health insurance, were merged with the medical conditions file, which contains information on self-reported medical conditions and *International Classification of Diseases, Ninth Revision, Clinical Modification (ICD*-*9*-*CM)* diagnosis codes, from 2006 to 2015. For ease of analysis and reporting, data were pooled into 2-year cycles as follows: 2006-2007, 2008-2009, 2010-2011, 2012-2013, and 2014-2015. Person-level weight adjustments were made to reflect the mean annual population size and expenditures for the 2 years per cycle.^26^

**Figure 1.**
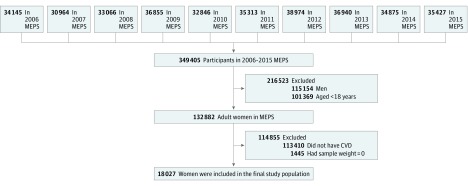
Flow Diagram of Patient Selection Process CVD indicates cardiovascular disease; MEPS, Medical Expenditure Panel Survey.

For this analysis, we included women who had a self-reported and/or *ICD-9-CM* diagnosis of CVD (*ICD-9-CM* codes for coronary artery disease [410, 413, and 414], stroke [433-437], heart failure [428], cardiac dysrhythmias [427], and/or peripheral arterial disease [440, 443, and 447]), with positive sampling weights (final survey person-weight >0 for national representativeness, ie, individuals with a weight of 0 were excluded) (Figure 1). As recommended by the Department of Health and Human Services, this study was exempt from institutional review board approval and informed consent because the MEPS consists of deidentified, publicly available data. This study is reported in accordance with the Consolidated Standards of Reporting Trials (CONSORT) reporting guideline.

### Study Variables

#### Physical Activity Classification

An individual’s response to the item on the self-administered questionnaire, “Do you now spend half an hour or more in moderate to vigorous PA, at least five times a week?”^27^ was used to analyze PA. As described in the MEPS glossary, “Moderate physical activity causes only light sweating or a slight or moderate increase in breathing or heart rate and would include activities such as fast walking, raking leaves, mowing the lawn, or heavy cleaning. Vigorous physical activity causes heavy sweating or large increases in breathing or heart rate and would include activities such as running, race walking, lap swimming, aerobics classes, or fast bicycling.”^28^ Individuals who responded yes to the PA question were classified as having optimal PA, and those who answered no were classified as having suboptimal PA. The trends in the responses to the PA question were tracked during a 10-year period to evaluate changes in self-reported levels of PA.

#### Participant Characteristics and Predictors of Suboptimal PA

We considered several factors in the evaluation of PA trends, including time, age, race/ethnicity, income level, education level, insurance status, geographic region, and comorbid conditions. Race/ethnicity was self-reported and categorized as non-Hispanic white, African American, Asian, or Hispanic. Based on the federal poverty level (FPL), family income was divided into 4 categories: high income (≥400% of FPL), middle income (200%-400% of FPL), low income (125%-200% of FPL), and poor or very low income (<125% of FPL).^23^ Education was labeled as less than high school, high school/general educational development or its equivalent, and at least some college. Marital status was labeled as married, divorced/widowed/separated, or never married. Age of respondents was categorized into 4 groups: 18 to 39 years, 40 to 64 years, 65 to 74 years, and 75 years or older. There were 4 categories for health insurance: private, uninsured, Medicaid, and Medicare. Geographical regions were divided into Northeast, Midwest, South, and West. The Charlson Comorbidity Index^29,30^ was used to estimate an individual’s comorbidity burden. In this study, the Charlson Comorbidity Index was modified to exclude the cardiovascular components.

#### Health Care Expenditure

Data for all health care expenditures in the MEPS include spending from out-of-pocket and all-payer groups from hospitalizations, medications, outpatient, and emergency department visits, and other medical expenditures incurred, such as vision aid and home health care. Participants reported all annual medical expenditures and the sources of payment. A follow-up survey was performed with the health care professionals and pharmacies to improve the accuracy of the data collected.^24^ We used the gross domestic product deflator to adjust all health care expenditures to 2015 US dollars.

### Statistical Analysis

The analyses for this project were conducted in August 2018. Stata version 14 (StataCorp) was used for statistical analyses. The svyset command in Stata was used to declare the data as a survey data set and the svy: proportion command to provide estimates of the proportion of the study population (n = 18 027 MEPS participants, representing approximately 19.5 million US women with CVD) based on their economic and sociodemographic characteristics per cycle. A weighted multivariable logistic regression was performed to determine the associations of sociodemographic predictor variables (cycle, age group, race/ethnicity, income level, education level, insurance status, marital status, and geographic region) with suboptimal PA levels, after adjusting for the Charlson Comorbidity Index. Odds ratios (ORs) and 95% CI were reported. When time was examined as a predictor of suboptimal PA, the 2006-2007 cycle was used as the reference cycle. A χ^2^ test for trend was used to assess the proportional differences in patient-reported suboptimal PA during cycles. *P* values were 2-tailed, and *P* values less than .05 were considered statistically significant.

As most health care expenditure data are seen in only a fraction of the population, 2-part models were used to model health care expenditures, which are a product of the probability that an individual incurred any health care cost. The 2-part model consists of a first part, using the probit command,^31,32^ and second part, using the generalized linear model command with γ distribution, to obtain mean per capita expenditures. A modified Park test was used to determine the distribution of the generalized linear model based on the most likely appropriate variance function, which considered γ, Gaussian, and inverse Gaussian distributions.^33^ Total expenditures were calculated using the post command after the 2-part models.^32^ We reported the total and out-of-pocket expenditures by cycle to estimate the trends in health care expenditure among women with CVD, stratified by suboptimal vs optimal PA, during the study.

## Results

### Baseline Characteristics

A total of 18 027 female MEPS participants were included in this study (Figure 1). The results are weighted to provide estimates for approximately 19.5 million adult women in the United States living with CVD (mean [SD] age, 60.4 [16.9] years). There were no differences observed in the sociodemographic characteristics of participants during the study (Table 1). Non-Hispanic white participants were 77.5% (95% CI, 75.9%-78.9%) of the study population. The study population consisted of 47.1% (95% CI, 45.5%-48.8%) of participants 65 years or older, 40.8% (95% CI, 39.5%-42.2%) of participants aged 40 to 64 years, and 12.1% (95% CI, 11.1%-12.9%) of participants younger than 40 years. The plurality of participants was in the high-income category (31.9%; 95% CI, 30.6%-33.3%), lived in the South (39.5%; 95% CI, 37.8%-41.3%), was married (45.5%; 95% CI, 43.9%-47.1%), and was enrolled in private health insurance (38.6%; 95% CI, 37.1%-40.2%) (Table 1).

**Table 1.** Characteristics of US Women With Cardiovascular Disease^a^

Characteristic	Prevalence, % (95% CI)					
	2006-2007	2008-2009	2010-2011	2012-2013	2014-2015	Total
Age, y						
18-39	9.7 (8.4-11.2)	13.2 (11.4-15.3)	11.8 (10.3-13.5)	12.6 (11.2-14.2)	12.1 (10.8-13.6)	12.1 (11.1-12.9)
40-64	39.5 (36.9-42.1)	41.6 (39.3-44.0)	40.9 (38.2-43.6)	40.9 (38.4-43.4)	40.9 (38.6-43.4)	40.8 (39.5-42.2)
65-74	19.7 (17.8-21.9)	19.2 (17.3-21.2)	19.7 (17.8-21.6)	18.9 (17.2-20.8)	21.4 (19.8-23.1)	19.8 (18.9-20.8)
≥75	31.1 (28.7-33.6)	26.0 (23.6-28.6)	27.6 (25.4-29.9)	27.6 (25.2-30.1)	25.6 (23.3-27.9)	27.3 (25.9-28.8)
Race/ethnicity						
Non-Hispanic white	78.9 (76.8-80.9)	78.9 (76.8-80.8)	77.3 (75.1-79.3)	77.2 (75.1-79.2)	75.6 (73.1-77.9)	77.5 (75.9-78.9)
African American	12.4 (10.8-14.2)	11.7 (10.3-13.2)	12.3 (10.7-14.1)	12.2 (10.5-14.0)	12.4 (10.8-14.1)	12.2 (11.0-13.4)
Asian	2.1 (1.5-2.9)	1.9 (1.2-2.9)	2.3 (1.7-3.1)	2.3 (1.8-3.1)	2.8 (2.0-3.9)	2.3 (1.8-2.9)
Hispanic	6.6 (5.6-7.8)	7.6 (6.4-8.9)	8.1 (6.8-9.7)	8.3 (6.9-9.9)	9.2 (7.8-10.7)	8.0 (7.1-9.1)
Insurance status						
Private	47.1 (43.9-50.2)	44.8 (41.9-47.6)	37.8 (35.1-40.6)	34.3 (31.6-37.1)	34.8 (32.5-37.2)	38.6 (37.1-40.2)
Uninsured	6.7 (5.5-7.9)	9.6 (8.3-11.2)	7.5 (6.4-8.8)	7.4 (6.3-8.7)	4.3 (3.5-5.4)	6.9 (6.3-7.6)
Medicaid	7.7 (6.6-8.9)	9.3 (7.9-10.8)	11.1 (9.6-12.8)	12.0 (10.6-13.6)	14.2 (12.7-15.8)	11.4 (10.5-12.3)
Medicare	38.5 (35.6-41.6)	36.3 (33.4-39.4)	43.6 (40.7-46.4)	46.3 (43.6-49.0)	46.7 (44.1-49.3)	43.1 (41.4-44.8)
Education level						
<High school	24.3 (22.4-26.4)	22.9 (21.0-24.9)	23.8 (21.4-26.4)	20.3 (18.4-22.2)	17.2 (15.5-19.1)	21.6 (20.4-22.8)
High school or GED equivalent	60.4 (57.9-62.8)	61.9 (59.2-64.4)	58.8 (55.7-61.8)	37.3 (34.9-39.6)	39.7 (37.3-42.2)	51.5 (50.1-52.9)
At least some college	15.3 (13.4-17.3)	15.2 (13.5-17.0)	17.4 (15.4-19.6)	42.5 (40.0-44.9)	43.1 (40.5-45.6)	26.9 (25.7-28.2)
Income strata						
High	35.3 (32.8-37.8)	31.7 (29.5-33.9)	29.6 (26.9-32.3)	30.4 (27.9-33.0)	33.3 (30.8-35.9)	31.9 (30.6-33.3)
Middle	27.1 (25.0-29.3)	29.3 (27.5-31.2)	30.6 (28.6-32.7)	27.6 (25.5-29.7)	27.7 (25.8-29.6)	28.5 (27.5-29.5)
Low	17.3 (15.7-19.0)	17.4 (15.9-18.9)	17.1 (15.5-18.8)	18.2 (16.5-20.1)	16.2 (14.6-17.9)	17.2 (16.4-18.1)
Very low or poor	20.3 (18.7-21.9)	21.6 (19.9-23.4)	22.7 (21.0-24.5)	23.8 (21.9-25.9)	22.8 (21.1-24.8)	22.4 (21.4-23.4)
Geographic region						
Northeast	18.9 (16.9-21.1)	18.4 (16.2-20.9)	18.5 (16.6-20.5)	17.7 (15.7-19.8)	17.7 (15.8-19.7)	18.2 (16.9-19.5)
Midwest	23.5 (21.2-25.9)	23.3 (20.8-25.9)	23.4 (21.1-25.9)	21.7 (19.5-23.9)	22.9 (20.5-25.5)	22.9 (21.5-24.4)
South	39.8 (36.9-42.7)	38.8 (35.9-41.8)	38.3 (35.8-40.9)	41.2 (38.5-44.1)	39.3 (36.3-42.5)	39.5 (37.8-41.3)
West	17.8 (15.5-20.4)	19.5 (17.3-21.8)	19.8 (17.8-22.0)	19.4 (17.6-21.3)	20.1 (17.4-23.1)	19.4 (18.0-20.9)
Marital status						
Married	45.9 (43.5-48.4)	45.7 (43.3-48.2)	44.8 (41.9-47.7)	44.4 (41.7-47.2)	46.6 (43.7-49.5)	45.5 (43.9-47.1)
Divorced, widowed, or separated	45.7 (43.1-48.2)	43.1 (40.6-45.7)	44.4 (41.4-47.3)	43.6 (40.9-46.3)	41.7 (39.3-44.2)	43.6 (42.1-45.1)
Never married	8.4 (7.2-9.9)	11.2 (9.6-12.9)	10.8 (9.3-12.5)	12.0 (10.6-13.5)	11.7 (10.2-13.3)	10.9 (10.1-11.8)

Abbreviation: GED, general educational development.

^a^ All percentages are weighted to provide estimates for approximately 19.5 million women in the United States.

### Trends in Suboptimal PA Among Women With CVD

**Figure 2.**
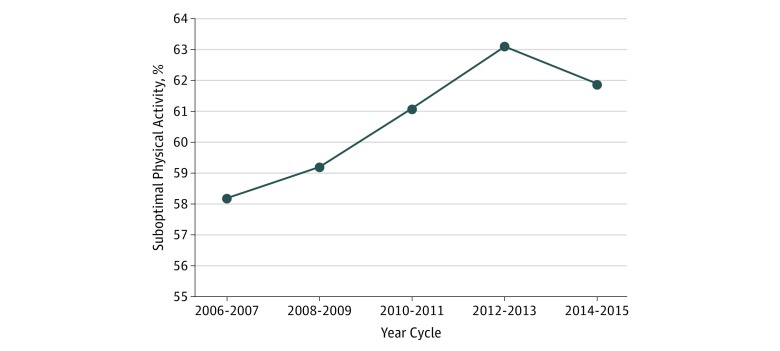
Trend in Estimated Suboptimal Physical Activity Among US Women With Cardiovascular Disease All percentages are weighted to provide estimates for approximately 19.5 million adult women in the United States. The proportion of women with cardiovascular disease with suboptimal physical activity levels increased from 58.2% in 2006-2007 to 61.9% in 2014-2015 (*P* = .004).

More than half of women with CVD reported suboptimal PA, and proportions with suboptimal PA increased during the study, from 58.2% (95% CI, 55.9%-60.5%) in 2006-2007 to 61.9% (95% CI, 59.7%-64.2%) in 2014-2015 (*P* = .004) (Figure 2). eFigures 1-5 in the Supplement show the trends of suboptimal PA through time by subgroups. The increase in proportion of women with suboptimal PA was most notable among those aged 40 to 64 years (53.4% [95% CI, 49.9%-56.9%] in the 2006-2007 cycle to 60.6% [95% CI, 57.0%-64.1%] in the 2014-2015 cycle; *P* = .01) (eFigure 1 in the Supplement), of African American race (55.5% [95% CI, 50.3%-60.6%] in the 2006-2007 cycle to 67.2% [95% CI, 63.6%-70.6%] in the 2014-2015 cycle; *P* < .001) (eFigure 2 in the Supplement), enrolled in private health insurance (44.6% [95% CI, 40.8%-48.6%] in the 2006-2007 cycle to 55.1% [95% CI, 50.8%-59.4%] in the 2014-2015 cycle; *P* < .001) (eFigure 3 in the Supplement), earning a high income (48.8% [95% CI, 44.8%-52.7%] in the 2006-2007 cycle to 59.5% [95% CI, 55.1%-63.8%] in the 2014-2015 cycle; *P* < .001) (eFigure 4 in the Supplement), and with at least some college (45.3% [95% CI, 39.4%-51.4%] in the 2006-2007 cycle to 58.3% [95% CI, 54.5%-61.9%] in the 2014-2015 cycle; *P* < .001)(eFigure 5 in the Supplement). Among the Medicare population, the proportion of women reporting suboptimal PA decreased from 71.4% (95% CI, 67.6%-74.8%) in the 2006-2007 cycle to 65.8% (95% CI, 62.7%-68.8%) in the 2014-2015 cycle (*P* = .04) (eFigure 3 in the Supplement). The subgroups with the highest proportions of suboptimal PA were women 75 years and older, of Hispanic or African American race/ethnicity, enrolled in Medicaid or Medicare insurance, from low- or very low-income strata, and with an education level of less than high school (eFigures 1-5 in the Supplement).

### Factors Associated With Suboptimal PA Among Women With CVD

Table 2 shows the sociodemographic factors associated with suboptimal PA adjusted for each other and for the modified Charlson Comorbidity Index. Compared with women aged 18 to 39 years, women aged 40 to 64 years (OR, 1.49; 95% CI, 1.23-1.80) and those 75 years and older (OR, 1.48; 95% CI, 1.03-2.12) had higher odds of reporting suboptimal PA. As income decreased, there was a stepwise increase in the odds of reporting suboptimal PA, (very low income vs high income: OR, 1.51; 95% CI, 1.27-1.80). Compared with non-Hispanic white participants, African American participants (OR, 1.22; 95% CI, 1.08-1.38) and Hispanic participants (OR, 1.33; 95% CI, 1.13-1.58) had higher odds of reporting suboptimal PA. Participants enrolled in Medicaid (OR, 1.39; 95% CI, 1.16-1.69) or Medicare (OR, 1.89; 95% CI, 1.38-2.60) had higher odds of reporting suboptimal PA compared with women enrolled in private health insurance plans. Women with higher levels of education had lower odds of reporting suboptimal PA than those with lower education levels (at least some college vs less than high school: OR, 0.81; 95% CI, 0.68-0.96).

**Table 2.** Odds Ratio for Suboptimal Physical Activity Among Women With Cardiovascular Disease

Variable	Odds Ratio (95% CI)^a^
Cycle	
2006-2007	1 [Reference]
2008-2009	1.05 (0.91-1.22)
2010-2011	0.95 (0.80-1.13)
2012-2013	1.29 (1.08-1.54)^b^
2014-2015	1.18 (1.00-1.39)
Age, y	
18-39	1 [Reference]
40-64	1.49 (1.23-1.80)^b^
65-74	1.25 (0.87-1.78)
≥75	1.48 (1.03-2.12)^b^
Income strata	
High	1 [Reference]
Middle	1.09 (0.93-1.26)
Low	1.29 (1.07-1.56)^b^
Very low or poor	1.51 (1.27-1.80)^b^
Race/ethnicity	
Non-Hispanic white	1 [Reference]
African American	1.22 (1.08-1.38)^b^
Asian	0.92 (0.65-1.29)
Hispanic	1.33 (1.13-1.58)^b^
Health insurance	
Private	1 [Reference]
Uninsured	1.15 (0.92-1.44)
Medicaid	1.39 (1.16-1.69)^b^
Medicare	1.89 (1.38-2.60)^b^
Education level	
<High school	1 [Reference]
High school or GED equivalent	0.92 (0.79-1.07)
At least some college	0.81 (0.68-0.96)^b^
Marital status	
Married	1 [Reference]
Divorced, widowed, or separated	0.96 (0.83-1.10)
Never married	0.91 (0.74-1.16)
Geographic region	
Northeast	1 [Reference]
Midwest	0.84 (0.69-1.02)
South	1.07 (0.89-1.28)
West	0.83 (0.69-0.99)^b^

Abbreviation: GED, general educational development.

^a^ Odds ratios were adjusted for cycle, age, race/ethnicity, health insurance, education level, income level, geographic region, marital status, and modified Charlson Comorbidity Index.

^b^ Results are statistically significant, *P* < .05.

### Trends in Health Care Expenditure

**Figure 3.**
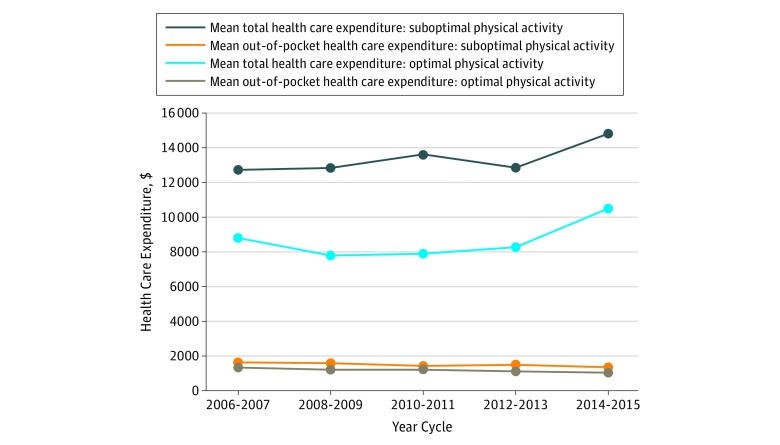
Trends in Estimated Health Care Expenditures Among US Women With Cardiovascular Disease, Based on Self-reported Physical Activity All percentages are weighted to provide estimates for approximately 19.5 million adult women in the United States.

Figure 3 shows the national trends in health care expenditure among US women with CVD based on their self-report of PA from 2006 to 2015. An increase in the total mean health care expenditure was demonstrated during the study. This increased financial burden was greater in those reporting suboptimal PA compared with women with CVD who reported meeting the PA recommendation. In the 2006-2007 cycle, the mean per capita total health care expenditure among women with CVD reporting suboptimal PA was $12 724 (95% CI, $11 627-$13 821), and this amount increased significantly in the 2014-2015 cycle, with a mean per capita health care expenditure of $14 820 (95% CI, $13 521-$16 119; *P* < .001). For women with optimal PA, their total expenditures also increased from $8811 (95% CI, $7750-$9872) in 2006-2007 to $10 504 (95% CI, $8845-$12 163) in 2014-2015 but remained less than for women with suboptimal PA throughout the whole period. Conversely, the mean out-of-pocket spending per capita decreased from $1643 (95% CI, $1506-$1780) in 2006-2007 to $1347 (95% CI, $1230-$1463) in 2014-2015 (*P* < .001) among those reporting suboptimal PA and from $1334 (95% CI, $1232-$1437) in 2006-2007 to $1040 (95% CI, $918-$1163) in 2014-2015 (*P* < .001) among those reporting optimal PA.

## Discussion

Using nationally representative survey data spanning 10 years, we found that more than half of all women with CVD reported suboptimal PA levels, and there was an increased proportion of women reporting suboptimal PA in each subsequent period, with the exception of 2014-2015. This was particularly pronounced among older women and women of African American or Hispanic race/ethnicity. Additionally, women with CVD from low- or very low-income strata (compared with high-income strata), enrolled in Medicaid or Medicare insurance (compared with private insurance), and with less than high school education (compared with at least some college) were more likely to have suboptimal PA. Mean total health care expenditures among women with CVD also increased through time, and expenditures were higher for women with suboptimal PA compared with optimal PA.

The benefits of PA among high-risk individuals, such as those with CVD, have been widely reported.^11,34^ The economic burden of CVD and how it may be reduced by meeting PA guidelines have also been previously described in general populations of men and women.^15,16,17,18,19,20^ However, to our knowledge, prior studies specifically focusing on PA in women with CVD are sparse, particularly for assessing trends through time using more contemporary data and examining vulnerable subgroups. By identifying subgroups of women with CVD who are at the greatest risk of suboptimal PA in our study, targeted intervention strategies can be implemented to optimize PA for secondary prevention and reduction in health care costs. For example, our study found that women in midlife (aged 40-64 years) were at greater risk of suboptimal PA compared with younger women. Although the reason for this observation is unknown, it is likely multifactorial. This may be because of the weight changes associated with menopausal transition or other changes in life status. Increasing awareness among these individuals, as well as developing strategies to optimize PA among this subgroup, may reduce the financial burden and improve cardiovascular morbidity and mortality among this population.

Survivors of myocardial infarction remain at higher risk of repeated cardiovascular events than the general population.^35^ A 2016 study^36^ of privately insured survivors of myocardial infarction revealed increased health care costs and an elevated risk of repeated cardiovascular events; similar results were found in an elderly population enrolled in Medicare who remained alive 1 year after myocardial infarction.^37^ Therefore, the importance of secondary prevention efforts, including increasing PA among individuals with CVD, cannot be overemphasized. Cardiac rehabilitation is an important venue to facilitate the optimization of PA among patients with CVD as part of comprehensive secondary prevention management.^34^ Despite this, the proportion of patients referred to these programs is low, especially for women.^38,39,40^ Additionally, it has been observed that certain patient populations, particularly older patients, racial/ethnic minorities, and those with low socioeconomic status, have even lower referral rates.^34^ The factors we found to be associated with suboptimal PA in our study are closely aligned with the factors associated with low cardiac rehabilitation referral rates described in other studies. An exercise-based cardiac rehabilitation program is not the only way to achieve PA in high-risk patients, but it is an important way to achieve PA targets after a CVD event and, hopefully, to encourage continued engagement in PA long after discharge from the program. If referral, enrollment, and participation in cardiac rehabilitation are optimized in women,^39,40^ particularly among these subgroups, it may improve long-term rates of favorable PA, although we could not assess this within our analyses.

Other ways to improve PA among women with CVD include assessing PA as a vital sign at every clinical encounter, with positive reinforcement by physicians and other health care professionals on the health benefits of PA—benefits that extend well beyond the cardiovascular system.^7^ Individuals with the least PA may benefit from even modest increases, while there may be additional benefits with even more PA than the recommended amount.^7^ Partnerships and group sessions can be organized for women with CVD to encourage each other to optimize their PA levels. Women with CVD could be advised to keep weekly exercise logs or use fitness-tracking smartphone apps that could be brought to follow-up outpatient visits. Mobile health-tracking technologies may also help facilitate PA in concert with other drivers of behavioral health changes.^41^

Overall in the US population, an estimated $117 billion in annual health care costs are attributed to not meeting the recommended PA guidelines.^7^ Although not limited to women, a prior study by Wang et al^19^ using 1996 MEPS data found higher medical expenditure among persons with CVD, of which approximately 13% was associated with lack of PA. Additionally, a 2017 study of 2012-2013 MEPS data^42^ of pharmaceutical expenditures of adults with CVD also showed significantly higher costs associated with suboptimal PA. Our study found that women with CVD who reported suboptimal PA levels had increased mean total health care expenditures than women who reported optimal PA through the entire period. We also found that total health care expenditures increased through time in both groups, particularly in the 2014-2015 cycle, which may be partly explained by increased health insurance coverage following the implementation of the Patient Protection and Affordable Care Act in 2014. While there was a slight decline in the mean out-of-pocket expenditure during the study, it is interesting to note that women with suboptimal PA still had slightly higher out-of-pocket costs than those reporting optimal PA. Another MEPS study using data from the 2012 MEPS,^20^ which evaluated patients with and without CVD, found that those with optimal PA had lower health care expenditures and resource utilization regardless of CVD status. These studies, combined with our 10-year findings among women with CVD, emphasize the importance of promoting PA to reduce the high economic burden associated with suboptimal PA levels in this high-risk secondary prevention population.

### Strengths and Limitations

Our study is strengthened by the design and execution of the MEPS, with its multilevel ascertainment of information obtained from survey participants.^43^ Also, an oversampling of racial/ethnic minorities was performed, making our results generalizable to all noninstitutionalized adult women with CVD in the United States. The large sample size enabled us to adequately characterize women with CVD and to further stratify the results by age, race/ethnicity, level of education, and other sociodemographic characteristics, as well as determine the health care expenditure trends during the study.

However, some limitations should be considered in the interpretation of our results. First, our observed levels of suboptimal PA may be underestimated because PA levels were self-reported. Also, owing to inadequate assessment of the degree of intensity in the self-administered questionnaire, we only dichotomized PA into those who engaged in optimal PA (≥30 minutes, ≥5 days per week) and those who did not, a group likely to include participants engaging in light or minimal PA as well as those who are not engaging in any PA. Third, underestimation of health expenditure costs in MEPS data has been reported by some studies^44,45^ and may have led to conservative estimates of the increasing costs associated with suboptimal PA in our study. Furthermore, we could not account for the timing of the CVD events and how they may have been associated with the level of PA. Fifth, although we adjusted for comorbidities, suboptimal PA could be a marker for increased CVD severity, which could not be assessed by our study design. However, further adjustments for self-perception of health status as a correlate for severity of illness were similar to our main findings but somewhat attenuated for a few of the factors (eTable in the Supplement).

## Conclusions

Using data from a nationally representative sample of US women living with CVD, we found that the proportion of women who report suboptimal PA was high and increased during a contemporary 10-year period. We also show that the proportions of women with suboptimal PA are higher among subgroups at greater risks as defined by age, race/ethnicity, and socioeconomic factors. Finally, we found that the economic burden associated with suboptimal PA among women with CVD was higher compared with women with optimal PA. Specific interventions targeting older women, those from lower socioeconomic status, and racial/ethnic minorities should be implemented to enable more women in these high-risk populations to fulfill the recommended PA guidelines for secondary prevention and achieve associated reduction in health care costs.

## Notes

Supplement. **eFigure 1.** National Trends in Suboptimal Physical Activity Levels Among Women With CVD, Stratified by Age Category **eFigure 2.** National Trends in Suboptimal Physical Activity Levels Among Women With CVD, Stratified by Race/Ethnicity **eFigure 3.** National Trends in Suboptimal Physical Activity Levels Among Women With CVD, Stratified by Health Insurance Status **eFigure 4.** National Trends in Suboptimal Physical Activity Levels Among Women With CVD, Stratified by Level of Income **eFigure 5.** National Trends in Suboptimal Physical Activity Levels Among Women With CVD, Stratified by Education Status **eTable.** Sensitivity Analysis After Further Adjusting for Self-Perception of Health
