# Supplementary material for: Trends and Costs Associated With Suboptimal Physical Activity Among US Women With Cardiovascular Disease
Source: JAMA Netw Open. 2019 Apr 12;2(4):e191977. doi: 10.1001/jamanetworkopen.2019.1977 (PMC6481430; doi:10.1001/jamanetworkopen.2019.1977)
Supplement: Supplement. — eFigure 1. National Trends in Suboptimal Physical Activity Levels Among Women With CVD, Stratified by Age Category eFigure 2. National Trends in Suboptimal Physical Activity Levels Among Women With CVD, Stratified by Race/Ethnicity eFigure 3. National Trends in Suboptimal Physical Activity Levels Among Women With CVD, Stratified by Health Insurance Status eFigure 4. National Trends in Suboptimal Physical Activity Levels Among Women With CVD, Stratified by Level of Income eFigure 5. National Trends in Suboptimal Physical Activity Levels Among Women With CVD, Stratified by Education Status eTable. Sensitivity Analysis After Further Adjusting for Self-Perception of Health [file jamanetwopen-2-e191977-s001.pdf]

Supplementary Online Content

Okunrintemi V, Benson E-MA, Tibuakuu M, et al. Trends and costs associated with suboptimal physical activity among US women with cardiovascular disease. *JAMA Netw Open*. 2019;2(4):e191977. doi:10.1001/jamanetworkopen.2019.1977

eFigure 1. National Trends in Suboptimal Physical Activity Levels Among Women With CVD, Stratified by Age Category

eFigure 2. National Trends in Suboptimal Physical Activity Levels Among Women With CVD, Stratified by Race/Ethnicity

eFigure 3. National Trends in Suboptimal Physical Activity Levels Among Women With CVD, Stratified by Health Insurance Status

eFigure 4. National Trends in Suboptimal Physical Activity Levels Among Women With CVD, Stratified by Level of Income

eFigure 5. National Trends in Suboptimal Physical Activity Levels Among Women With CVD, Stratified by Education Status

eTable. Sensitivity Analysis After Further Adjusting for Self-Perception of Health

This supplementary material has been provided by the authors to give readers additional information about their work.

© 2019 Okunrintemi V et al. *JAMA Network Open.*

eFigure 1. National Trends in Suboptimal Physical Activity Levels Among Women With CVD, Stratified by Age Category

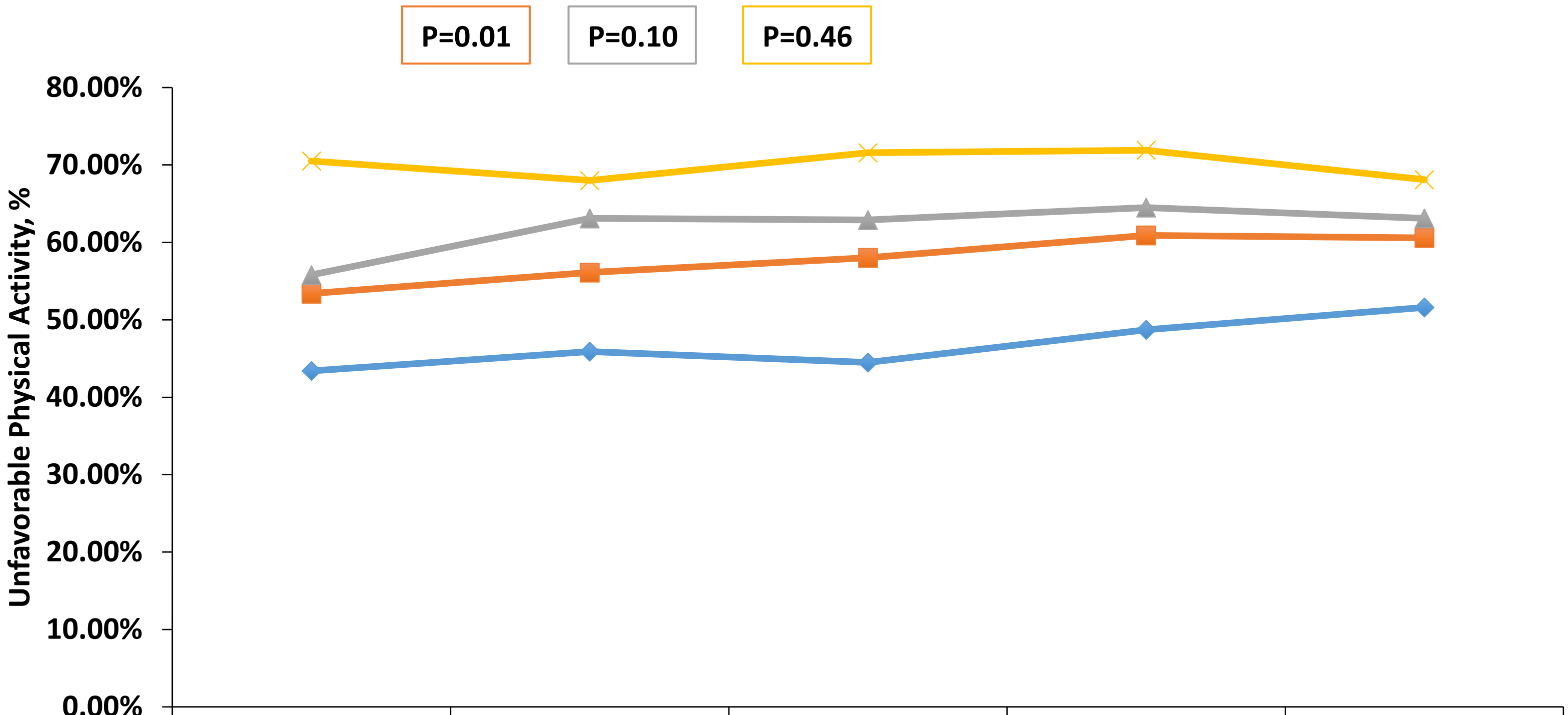

	2006 - 2007	2008 - 2009	2010 - 2011	2012 - 2013	2014 - 2015
18-39 years	43.40%	45.90%	44.50%	48.70%	51.60%
40-64 years	53.40%	56.10%	58.00%	60.90%	60.60%
65-74 years	55.80%	63.10%	62.90%	64.50%	63.10%
≥ 75 years	70.50%	68.00%	71.60%	71.90%	68.10%

© 2019 Okunrintemi V et al. *JAMA Network Open.*

eFigure 2. National Trends in Suboptimal Physical Activity Levels Among Women With CVD, Stratified by Race/Ethnicity

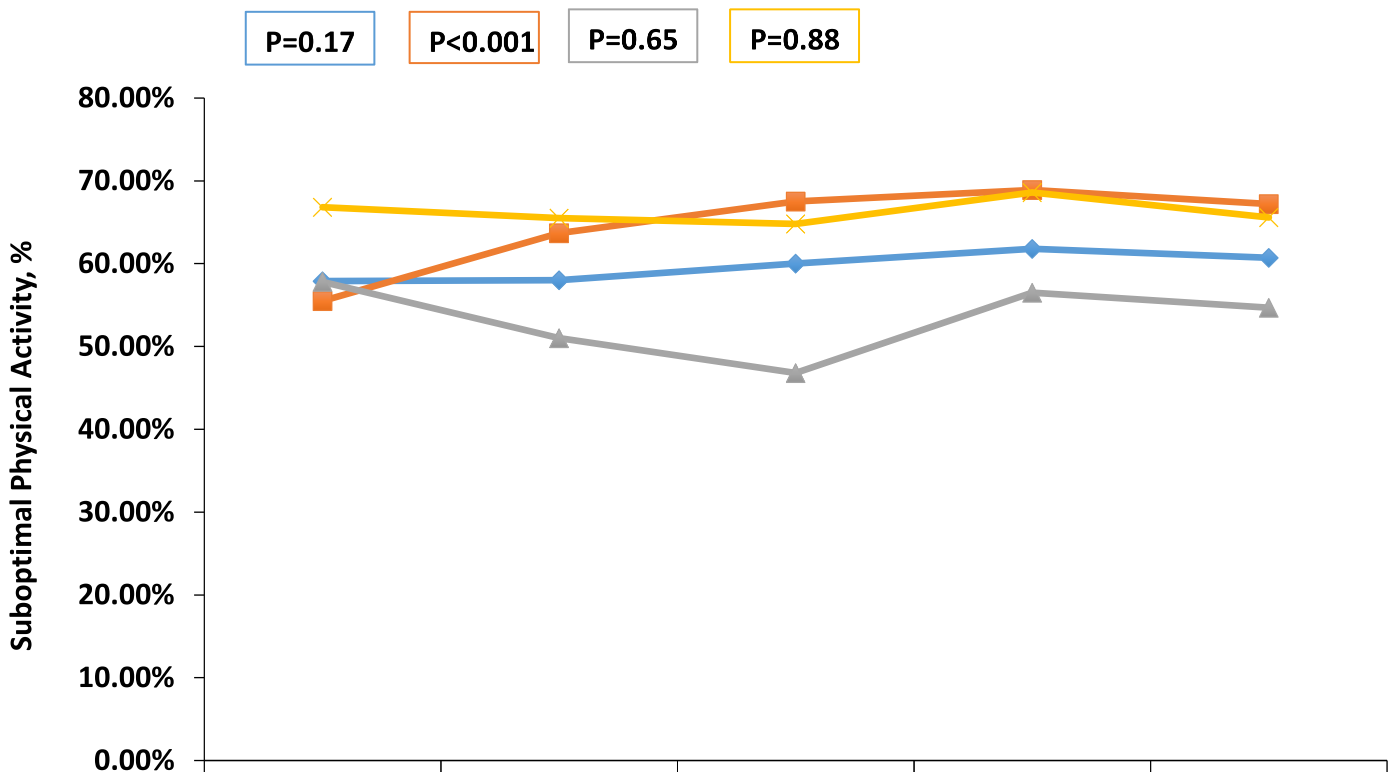

	2006 - 2007	2008 - 2009	2010 - 2011	2012 - 2013	2014 - 2015
Non-Hispanic white	57.90%	58.00%	60.00%	61.80%	60.70%
African-American	55.50%	63.70%	67.50%	68.90%	67.20%
Asian	57.80%	51.00%	46.80%	56.50%	54.70%
Hispanic	66.80%	65.50%	64.80%	68.60%	65.60%

© 2019 Okunrintemi V et al. *JAMA Network Open.*

eFigure 3. National Trends in Suboptimal Physical Activity Levels Among Women With CVD, Stratified by Health Insurance Status

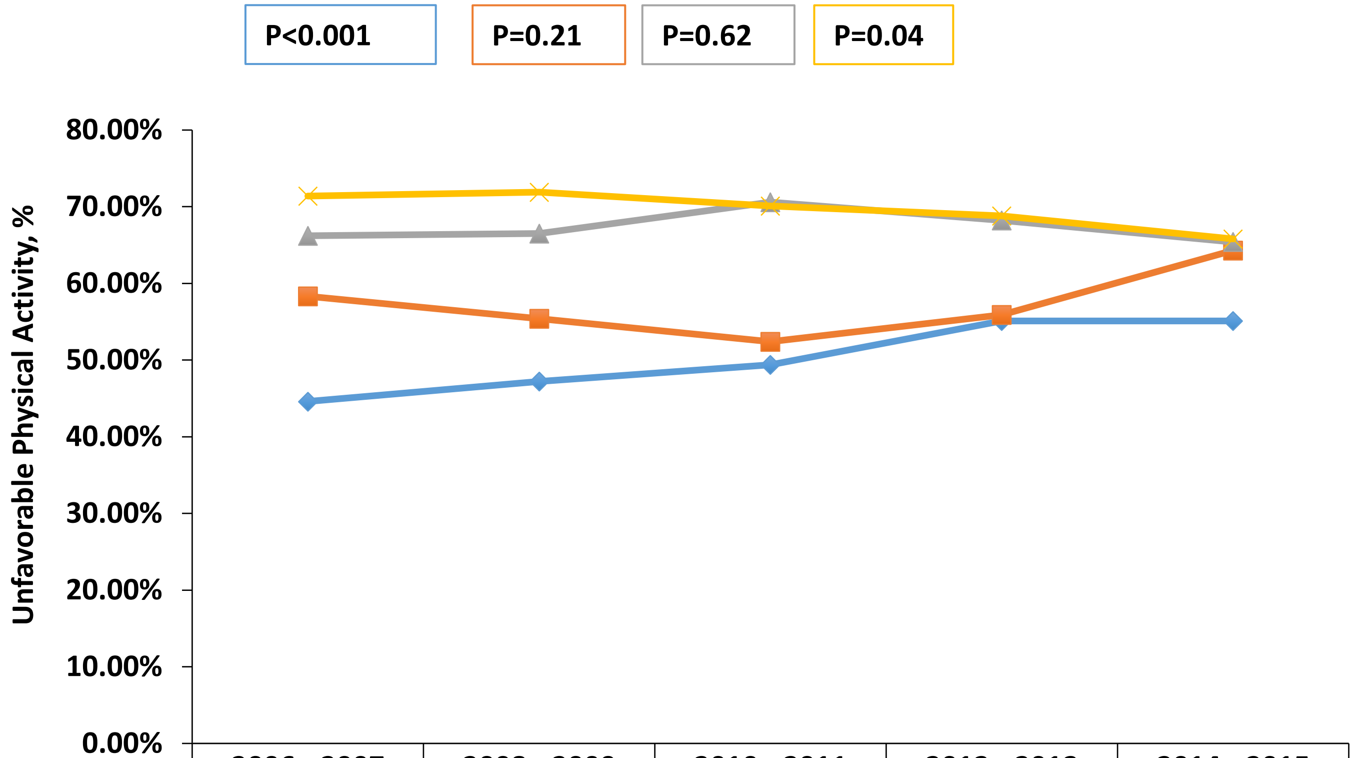

	2006 - 2007	2008 - 2009	2010 - 2011	2012 - 2013	2014 - 2015
Private	44.60%	47.20%	49.40%	55.10%	55.10%
Uninsured	58.30%	55.40%	52.40%	55.90%	64.30%
Medicaid	66.20%	66.50%	70.60%	68.20%	65.40%
Medicare	71.40%	71.90%	70.10%	68.80%	65.80%

© 2019 Okunrintemi V et al. *JAMA Network Open.*

eFigure 4. National Trends in Suboptimal Physical Activity Levels Among Women With CVD, Stratified by Level of Income

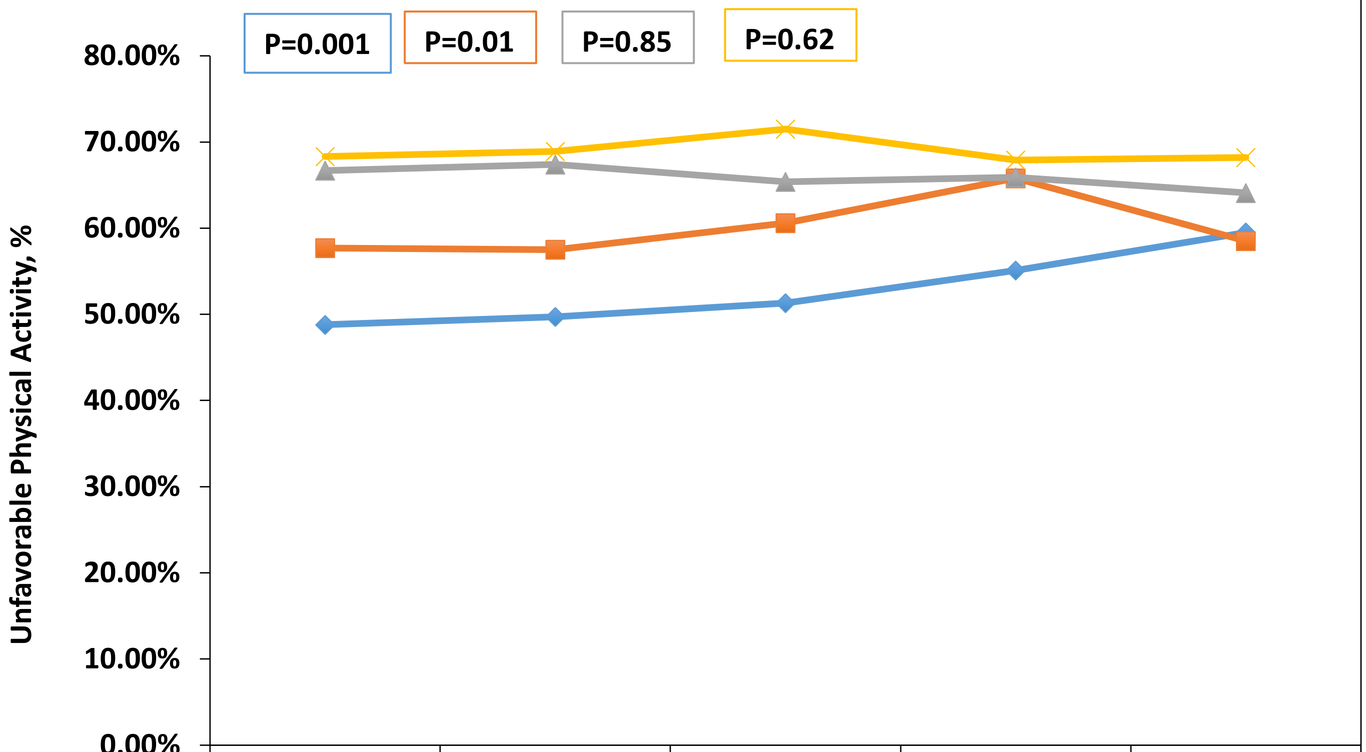

	2006 - 2007	2008 - 2009	2010 - 2011	2012 - 2013	2014 - 2015
High income	48.80%	49.70%	51.30%	55.10%	59.50%
Middle income	57.70%	57.50%	60.60%	65.80%	58.50%
Low income	66.70%	67.40%	65.40%	65.90%	64.10%
Poor/very low income	68.30%	68.90%	71.50%	67.90%	68.20%

© 2019 Okunrintemi V et al. *JAMA Network Open.*

eFigure 5. National Trends in Suboptimal Physical Activity Levels Among Women With CVD, Stratified by Education Status

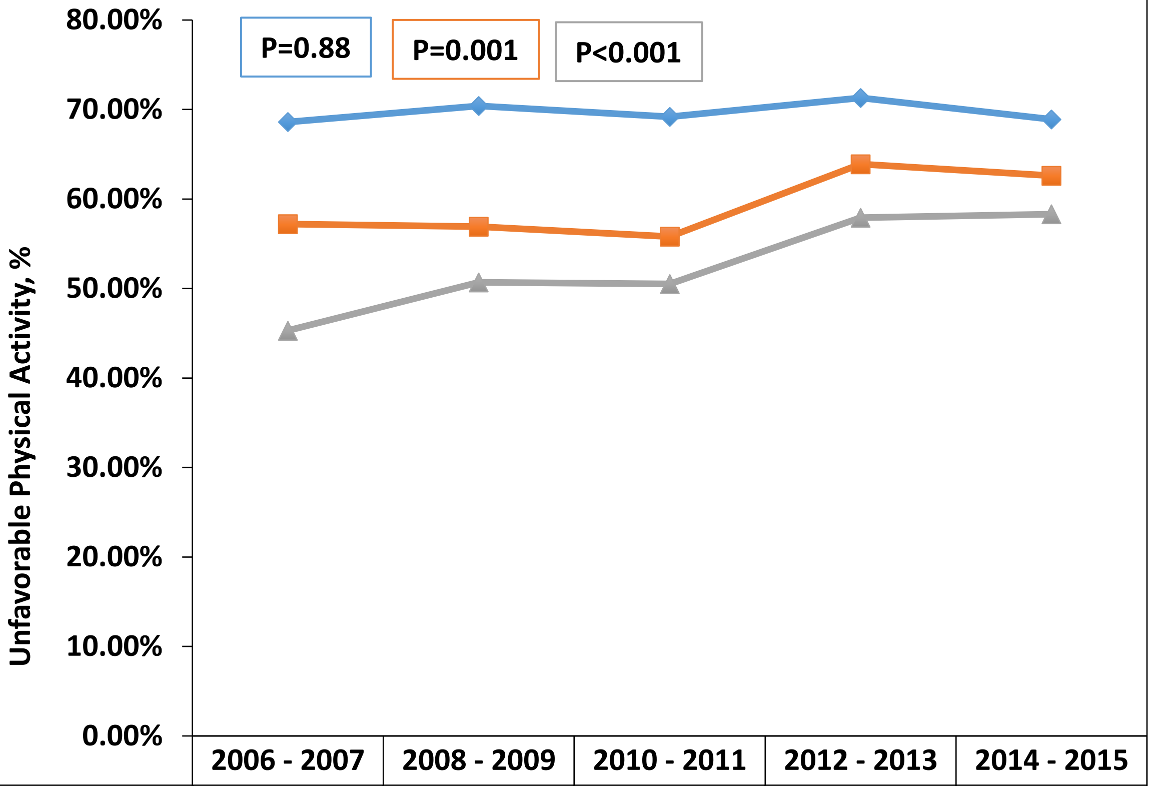

	2006 - 2007	2008 - 2009	2010 - 2011	2012 - 2013	2014 - 2015
less than high school	68.60%	70.40%	69.20%	71.30%	68.90%
High school/GED or its equivalent	57.20%	56.90%	55.80%	63.90%	62.60%
College or higher	45.30%	50.70%	50.50%	57.90%	58.30%

© 2019 Okunrintemi V et al. *JAMA Network Open.*

eTable. Sensitivity Analysis After Further Adjusting for Self-Perception of Health

	Likelihood of a US adult female to report suboptimal physical activity
Variables	*Odds ratios (95% CI)*
Cycle	
2006-2007	Ref
2008-2009	1.09 (0.93 – 1.27)
2010-2011	1.10 (0.94 – 1.28)
2012-2013	1.27 (1.09 – 1.48)
2014-2015	1.20 (1.02 – 1.41)
Age group (years)	
18 - 39	Ref
40 – 64	1.37 (1.16 – 1.62)
65 - 74	1.38 (0.98 – 1.96)
≥ 75	1.95 (1.39 – 2.73)
Level of income	
High income	Ref
Middle income	1.03 (0.90 – 1.17)
Low income	1.16 (0.98 – 1.36)
Very low income/poor	1.24 (1.06 – 1.44)
Race/ethnicity	
Non-Hispanic whites	Ref
African Americans	1.20 (1.07 – 1.35)
Asians	0.78 (0.57 – 1.04)
Hispanic	1.22 (1.04 – 1.43)
Health insurance	
Private	Ref
Uninsured	1.15 (0.85 – 1.23)
Medicaid	1.12 (0.96 – 1.31)
Medicare	1.32 (0.99 – 1.77)
Education	
Less than high school	Ref
High school/GED or its equivalent	0.89 (0.78 – 1.03)
College or higher	0.85 (0.72 – 1.00)
Marital status	
Married	Ref
Divorced/Widowed/Separated	0.94 (0.84 – 1.06)
Never married	0.99 (0.84 – 1.18)
Region	
Northeast	Ref
Midwest	0.88 (0.73 – 1.06)
South	1.02 (0.85 – 1.23)
West	0.88 (0.74 – 1.05)
Odds ratios were adjusted for cycle, age group, race/ethnicity, health insurance, education, income level, region, marital status, modified charlson comorbidity index, **self perception of health**	

© 2019 Okunrintemi V et al. *JAMA Network Open.*
